# *FON4* prevents the multi‐floret spikelet in rice

**DOI:** 10.1111/pbi.13083

**Published:** 2019-02-06

**Authors:** Deyong Ren, Qiankun Xu, Zhennan Qiu, Yuanjiang Cui, Tingting Zhou, Dali Zeng, Longbiao Guo, Qian Qian

**Affiliations:** ^1^ State Key Lab of Rice Biology China National Rice Research Institute Hangzhou China; ^2^ Agricultural Genomics Institute at Shenzhen Chinese Academy of Agricultural Sciences Shenzhen China

**Keywords:** spikelet morphogenesis, multi‐floret spikelet, spikelet meristem determinacy, CRISPR‐Cas9, rice (*Oryza sativa*), FON4

The spikelet is the specific unit of the grass inflorescence, and produces a variable number of florets (1–40) depending on whether the species displays determinate or indeterminate growth. Spikelet morphogenesis therefore plays an important role in crop yield; for example in wheat (*Triticum aestivum*) inflorescences with the indeterminate spikelet, the spikelet meristem is continuously active and produces a plethora of lateral floral meristems, leading to the formation of variable numbers of florets. In inflorescences of rice (*Oryza sativa*) and maize (*Zea mays*) with the determinate spikelet, the spikelet meristem produces the fixed numbers of lateral floral meristems, after which its fate is terminated as a terminal floral meristem, resulting in the formation of one or two florets. In rice, *MFS1*,* SNB*,* EG1*/*DF1*,* TOB1* and *OsMADS22* regulates the spikelet determinacy or indeterminacy (Lee *et al*., 2007; Ren *et al*., 2013). The loss of function of any of these genes induces the production of two complete florets or extra lemmas (secondary florets) within a single spikelet. In this study, we described a new mutant allele of *FON4*, named *fon4‐7*.

In the previous studies, the *fon4* mutant produced increased inner floral organs and elongated sterile lemmas with the indeterminate identity (Chu *et al*., 2006), whereas we confirmed that the *fon4‐7* mutant showed two florets and induced the reversion of sterile lemmas into lemmas (lateral florets). We discuss two possibilities for the formation of multi‐floret spikelet in rice, which may facilitate the breeding of rice cultivars with increased grain number per rice panicle.

**Figure 1:**
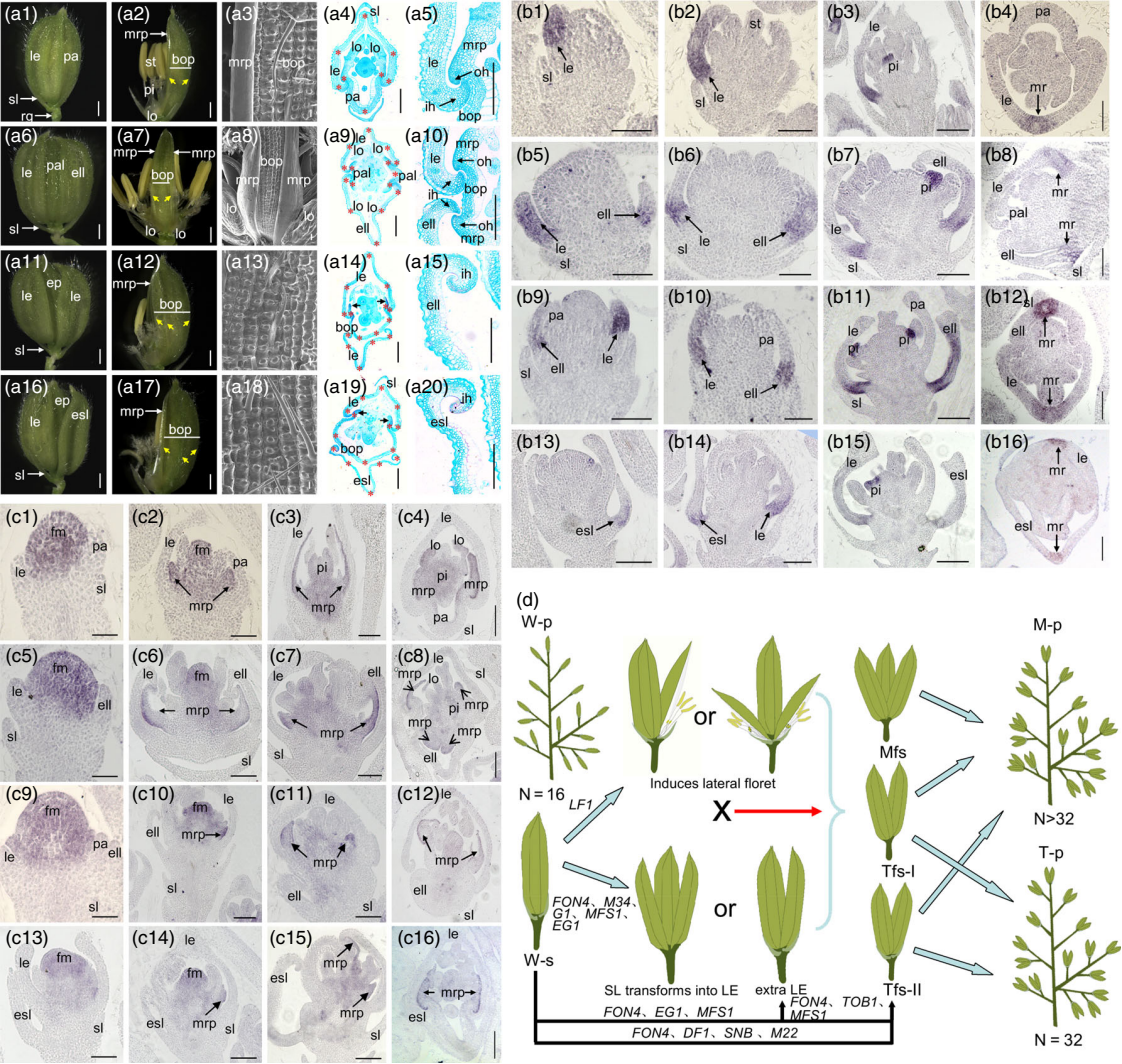
A, phenotypes investigations of spikelets in the wild type and *fon4‐7* mutant. A1 and A2, spikelet of the wild type. A3, epidermal surface of palea in the wild type. A4 and A5, histological observations of spikelet in the wild type. A6 and A7, *fon4‐7* spikelet with an extra lemma‐like organ, two reduced paleae and four lodicules. A8, epidermal surface of the reduced palea in A7. A9 and A10, histological analysis of an *fon4‐7* spikelet with an extra lemma‐like organ, two degenerated paleae and four lodicules. A11 and A12, *fon4‐7* spikelet with an extra lemma‐like organ and an enlarged palea. A13, epidermal surface of the extra lemma‐like organ in A12. A14 and A15, histological analysis of an *fon4‐7* spikelet with an extra lemma‐like organ and an enlarged palea. A16 and A17, *fon4‐7* spikelet with an elongated sterile lemma and an enlarged palea. A18, epidermal surface of the elongated sterile lemma in A17. A19 and A20, histological analysis of an *fon4‐7* spikelet with an elongated sterile lemma and an enlarged palea. B, expression of *DL* gene in the wild type and *fon4‐7* mutant. B1‐B4, wild‐type spikelet. B1, Sp4; B2, Sp5‐6; B3, Sp7, B4, Sp8. B5‐B8, *fon4‐7* spikelet with an extra lemma‐like organ and two reduced paleae. B5, Sp4; B6, Sp5‐6; B7, Sp7, B8, Sp8. B9‐B12, *fon4‐7* spikelet with an extra lemma‐like organ and an enlarged palea. B9, Sp4; B10, Sp5‐6; B11, Sp7, B12, Sp8. B13‐B16, *fon4‐7* spikelet with an elongated sterile lemma and an enlarged palea. B13, Sp4; B14, Sp5‐6; B15, Sp7, B16, Sp8. C, expression of *OsMADS6* gene in the wild type and *fon4‐7* mutant. C1‐C4, wild‐type spikelet. C1, Sp4; C2, Sp5‐6; C3, Sp7, C4, Sp8. C5‐C8, *fon4‐7* spikelet with an extra lemma‐like organ and two reduced paleae. C5, Sp4; C6, Sp5‐6; C7, Sp7, C8, Sp8. C9‐C12, *fon4‐7* spikelet with an extra lemma‐like organ and an enlarged palea. C9, Sp4; C10, Sp5‐6; C11, Sp7, C12, Sp8. C13‐C16, *fon4‐7* spikelet with an elongated sterile lemma and an enlarged palea. C13, Sp4; C14, Sp5‐6; C15, Sp7, C16, Sp8. D, model of hypothesized molecular design breeding and putative function of *FON4*. In the modle, *LF1* induces the lateral florets in the single rice spikelet with normal sterile lemma; *FON4*,*TOB1*,* M34*,*SNB*,* G1*,*MFS1*,*EG1/DF1* repress the formation of lemma‐like sterile lemma or extra lemma or two‐floret spikelet in the single rice spikelet with normal sterile lemma or without sterile lemma; *M22* induces the two‐floret spikelet in the single rice spikelet with normal sterile lemma. esl, elongated sterile lemma; ell, extra lemma‐like organ; le, lemma; lo, lodicule; st, stamen; pi, pistil; pa, palea; pal, palea‐like organ; rg, rudimentary glume; sl, sterile lemma; ep, enlarged palea; mrp, marginal regions of the palea; bop, body of the palea; oh, outward hook‐like structure; ih, inward hook‐like structure, fm, floral meristem; W‐p, wild‐type panicle; M‐p, panicle with multi‐floret spikelet; T‐p, panicle with two‐floret spikelet; W‐s, wild‐type spikelet; Mfs, multi‐floret spikelet; Tfs‐I, two‐floret spikelet without sterile lemma; Tfs‐II, two‐floret spikelet with sterile lemma; *M22*,* OsMADS22*;* M34*,* OsMADS34*. Black arrows represent mrp in A14 and A19. Yellow arrows and red stars represent vascular bundles in A. N indicates the floret number in panicle in D. “X” in D indicates the cross in breeding. Bars = 1000 mm in A1, A2, A6, A7, A11, A12, A16 and A17; 100 μm in A3‐A5, A8‐A10, A13‐A15, and A18‐A20; and 50 μm in B and C.

Rice strictly produces a pair of sterile lemmas and one floret per spikelet, each containing one lemma and one palea, two lodicules adjacent to the lemma, six stamens and a pistil (Figure 1A1–A5).

The group‐I *fon4‐7* spikelets developed extra lemma‐like organs, and two palea‐like organs (Figure 1A5–A10). Four lodicules were observed in these spikelets, two on either side of each lemma (Figure 1A7–A9). This phenotype indicated that these spikelets formed two florets. The group‐II spikelets produced a secondary floret containing a lemma‐like organ (Figure 1A11–A15), whereas the group‐III spikelets developed a terminal floret with a lemma‐like sterile lemma (lateral florets) (Figure 1A16–A20). Our detailed phenotypic observations revealed that all lemma‐like organs, lemma‐like sterile lemmas and enlarged body of the paleae had similar cell structures and vascular bundle numbers as the wild‐type lemmas (Figure 1A4, A14, A19). *OsMADS1*,* OsMADS14* and *OsMADS15*, were expressed in the typical lemmas, additional lemma‐like organs, elongated sterile lemmas and palea‐like organs of *fon4‐7* mutant. *DL* expression was also detected in the above organs except the palea‐like organs in the *fon4‐7* mutant. *OsMADS6* was expressed in the *fon4‐7* paleae and palea‐like organs. These changes in expression revealed that the extra lemma‐like organs and the elongated sterile lemmas of the *fon4‐7* spikelets had the lemma identity, and that the palea‐like organ was formed of a degraded palea. The increased expressions of *OsMADS2* and *OsMADS6* in the *fon4‐7* mutant may be due to the relative abundance of the corresponding organs of this mutant.

We monitored the development of early spikelets. At spikelet stage 4 (Sp4), some of the *fon4‐7* spikelets produced extra lemma‐like primordia and broad or narrow palea primordial. During stages Sp5 and Sp6, the *fon4‐7* spikelets formed higher numbers of stamens and maintained the growth of their defective palea. At Sp7 and Sp8, extra lemma‐like, palea‐like organs and elongated sterile lemmas were obviously observed in the *fon4‐7* spikelets, and the paleae were broader and had more than one bumped tops. No obvious differences of the sterile lemmas were found between the wild type and *fon4‐7* mutant at stages Sp4 to Sp7. At Sp8, the sterile lemmas of the *fon4‐7* mutant dramatically differentiated and were comparable in size to the wild‐type lemmas.

We next examined the expression patterns of floral organ identity genes using *in situ* hybridization. In the wild type, *DL* expression was detected in the lemma at stages Sp4 to Sp7, and in the midrib of the lemma at Sp8 (Figure 1B1–B4). In the *fon4‐7* mutant, *DL* expression was detected in the normal lemmas, extra lemma‐like organs and elongated sterile lemmas (Figure 1B5–B16). These results confirmed that the mutant spikelets indeed formed an additional lemma or underwent the homologous transformation of a sterile lemma into a lemma. During the Sp4 to Sp7 stages, *OsMADS6* expression in the *fon4‐7* mutant resembled that of the wild type, with transcription signals observed in the floral meristem, marginal regions of the palea (mrp), lodicule and pistil (Figure 1C1–C3, C5–C7, C9–C11, C11–C15). At Sp8, *fon4‐7* expressed this gene in the four mrps of the two palea‐like organs (C4, C8, C12, C16). These findings further indicated that the two palea‐like structures in the single *fon4‐7* spikelet were derived from normal paleae. The *OsMADS2* signals were detected in the stamens and the lodicules adjacent to the lemma in the wild type during stages Sp4 to Sp8; however, in group‐I *fon4‐7* spikelets, the transcripts of this gene were detected in the typical lodicules and extra lodicules at stages Sp4 to Sp7. At Sp8, the *OsMADS2* transcription signals were pronounced in the two lodicules on each side of the lemma in the individual *fon4‐7* spikelet, confirming that four lodicules were produced in the single *fon4‐7* spikelet containing two lemmas. These results supported the conclusion that two independent florets were formed in the single *fon4‐7* spikelet.

The *FON4* locus was narrowed between the markers M7 and M13. DNA sequencing led to the identification of an amino acid mutation in the *fon4‐7* mutant within the *Os11g38270*. A complementation assay showed that the *fon4‐7* phenotypes were completely rescued. The CRISPR/Cas9 knocked out *fon4‐8* and *fon4‐9* mutants exhibited the phenotypes that resembled the *fon4‐7* mutant. These results further supported that the *FON4* is the *Os11g38270* gene. Further investigations revealed that *fon4‐8* and *fon4‐9* induced a variable number of multi‐floret spikelet, suggesting that the different variation sites or genetic background in the *FON4* gene led to more and less multi‐floret spikelet.

About 41% of *fon4‐7* spikelets produced a typical lemma and an extra lemma, two palea‐like organs, increased stamens and pistils, and four lodicules in which each two on either side of each lemma, suggesting that two florets are formed. About 16% of *fon4‐7* spikelets bore normal florets with additional lemmas, which implied that these spikelets comprised a terminal floret and a secondary floret containing only the lemma. These findings suggest that the spikelet meristem determinacy was lost in the *fon4‐7* mutant. This phenotype of two florets or an extra lemma‐like organ (secondary floret) was also observed previously in the *tob1*,* mfs1*,* snb* and *eg1*/*df1* mutants, as well as the *snb*+*osids1* double mutant and *OsMADS22*‐overexpressing plants (Lee *et al*., 2007; Li *et al*., 2009; Ren *et al*., 2013, 2018; Sentoku *et al*., 2005; Tanaka *et al*., 2012). A recent study revealed that the mutation of *LF1* results in the formation of one or two fertile lateral florets (Zhang *et al*., 2017). These studies suggest that *FON4*,* LF1*,* TOB1*,* MFS1*,* SNB* and *OsMADS22* regulate the spikelet meristem determinacy, or their mutations or ectopic expressions induce the spikelet meristem indeterminacy. In wheat and oats (*Avena sativa*), the spikelet meristem exhibits indeterminacy and develops multiple florets, forming more than one seed per spikelet. These findings demonstrate that it is possible to generate two or more florets in each individual rice spikelet, raising the possibility of increasing the number of seeds produced per plant. Furthermore, we revealed that the *fon4* mutants produced a variable number of multi‐floret spikelet, indicating that the mutation of different nucleotides in *FON4* results in variable effects on its ability to control the spikelet determinacy, thereby producing more and less multi‐floret spikelet.

Previous studies have shown that the mutation of *G1*,* DF1*/*EG1* or *OsMADS34* caused the formation of lemma‐like sterile lemmas, whereas the mutation of *LF1* induced the formation of lateral florets without lemmas (Li *et al*., 2009; Lin *et al*., 2014; Yoshida *et al*., 2009; Zhang *et al*., 2017). These morphological findings strongly supported the three‐floret spikelet hypothesis (Zhang *et al*., 2017). The loss of *FON4*,* DF1*/*EG1*,* TOB1*,* MFS1* and *SNB* function causes the development of an extra lemma‐like organ or the formation of two florets within a single spikelet, potentially enabling the development of the residual spikelet meristems or transformation of determinacy into indeterminacy. These findings provide two possibilities for breeding rice cultivars with multi‐floret spikelet. One option is to cross the *lf1* mutant with our *fon4* mutant or other mutants (*m34*,* g1*,* mfs1* and *eg1*) that produce a lemma‐like sterile lemma or an extra lemma‐like organ within a single spikelet, with the aim of eventually generating cultivars with two‐ or three‐floret spikelet (Figure 1D). The other possibility is to identify different gene‐editing target sites in the genes (*FON4*,* DF1*/*EG1*,* TOB1*,* MFS1*,* SNB* and *M22*) involved in the regulation of spikelet meristem determinacy and indeterminacy (Figure 1D). Multi‐floret spikelet has the potential to increase grain number per panicle, thereby increasing rice yield.
